# Preparing for colorectal surgery: a qualitative study of experiences and preferences of patients in Western Canada

**DOI:** 10.1186/s12913-022-08130-y

**Published:** 2022-06-01

**Authors:** Rebecca Wang, Christopher Yao, Stanley H. Hung, Logan Meyers, Jason M. Sutherland, Ahmer Karimuddin, Kristin L. Campbell, Annalijn I. Conklin

**Affiliations:** 1 Faculty of Pharmaceutical Sciences, University of British Columbia, Vancouver, Canada; 2 Centre for Health Evaluation and Outcome Sciences, Providence Health Care Research Institute, Vancouver, Canada; 3 Department of Occupational Therapy, University of British Columbia, Vancouver, Canada; 4 Department of Physical Therapy, University of British Columbia, Vancouver, Canada; 5 School of Population and Public Health, Centre for Health Services and Policy Research, University of British Columbia, Vancouver, Canada; 6 Colorectal Surgery, St. Paul’s Hospital, Providence Health Care, and General Surgery Residency Training Program at the University of British Columbia, Vancouver, Canada

**Keywords:** Preoperative care, Prehabilitation, Colorectal cancer, Abdominal surgery, Quality improvement

## Abstract

**Objectives:**

The burden and costs of abdominal surgery for chronic conditions are on the rise, but could be reduced through self-management support. However, structured support to prepare for colorectal surgery is not routinely offered to patients in Canada. This study aimed to describe experiences and explore preferences for multimodal prehabilitation among colorectal surgery patients.

**Methods:**

A qualitative descriptive study using three focus groups (FG) was held with 19 patients who had a surgical date for abdominal surgery (April 2017-April 2018) and lived close (≤ 50 km radius) to a tertiary hospital in Western Canada (including a Surgical Lead for the British Columbia Enhanced Recovery After Surgery (ERAS) Collaborative). FGs were audio-taped and verbatim transcribed with coding and pile-and-sort methods performed by two independent reviewers, confirmed by a third reviewer, in NVivo v9 software; followed by thematic analysis and narrative synthesis.

**Results:**

Four themes emerged: support, informed decision-making, personalization of care, and mental/emotional health, which patients felt was particularly important but rarely addressed. Patient preferences for prehabilitation programming emphasised regular support from a single professional source, simple health messages, convenient access, and flexibility.

**Conclusions:**

There is an unmet need for structured preoperative support to better prepare patients for colorectal surgery. Future multimodal prehabilitation should be flexible and presented with non-medical information so patients can make informed decisions about their preoperative care and surgical outcomes. Healthcare providers have an important role in encouraging healthy lifestyle changes before colorectal surgery, though clearer communication and accurate advice on self-care, particularly mental health, are needed for improving patient outcomes.

**Supplementary Information:**

The online version contains supplementary material available at 10.1186/s12913-022-08130-y.

## Highlights

• Optimising preoperative care for abdominal surgery patients could improve outcomes.

• Patient experiences of preparing for colorectal surgery could inform future interventions, but patient-oriented research to improve quality of care is scarce.

• There is an unmet need for preoperative support in colorectal surgery.

• Future multimodal prehabilitation should incorporate patient preferences.

**Supplementary Information:**

The online version contains supplementary material available at 10.1186/s12913-022-08130-y.

## Introduction

Surgical treatment for colorectal conditions continues to be more common and costlier. Research on non-surgical methods for reducing complications [1] and perioperative care for improving outcomes is gaining prominence [2]. An important element of perioperative care includes ‘prehabilitation,’ a concept relatively new to abdominal surgery, and defined as the process of enhancing an individual’s physical functioning before surgery to improve their recovery from surgical stress [3]. There is burgeoning evidence that optimizing preoperative health is a promising approach to improving patient outcomes and reducing costs across the perioperative period among patients with colorectal cancer [4].

Much of the literature on preoperative care among abdominal surgery patients has focused on singular aspects of prehabilitation in cancer care [4, 5]. However, addressing multiple modifiable behavioural risk factors may provide stronger benefits to patients through synergistic effects on health [1, 4]. Considering multiple interdependent domains of health to improve patients’ surgical outcomes, particularly quality of life, is consistent with broader recommendations for effective chronic disease management and high-quality care [6]. Some recent literature is starting to explore the concept of multimodal prehabilitation in colorectal cancer [7], but the science regarding its effective elements is still nascent. Indeed, the only first international randomized control trial for multimodal prehabilitation in colorectal cancer surgical patients is underway [8]. This type of complex intervention aims to address multiple modifiable lifestyle factors (e.g. exercise, smoking, diet) during the pre-operative period (extending the peri-operative Enhanced Recovery After Surgery, ERAS).

While there is a general health services research gap on optimising care for abdominal surgery patients, far less is known about patient experiences of preparing for abdominal surgery during the preoperative period [9]. Moreover, current multimodal prehabilitation aims to recover only short-term physical functioning [4, 8], rather than improve patient-reported outcomes such as quality-of-life. Recent qualitative research among colorectal cancer survivors in Australia indicates a desire for support and simple health messages [10], although the information and support needs may differ for patients with non-cancer colorectal conditions. Active support from a health coach, moreover, has been shown to increase exercise and improve physical and mental health in British patients with chronic disease [11], and may be relevant to colorectal surgery. Current prehabilitation programs mostly provide patients with colorectal cancer with aerobic and resistance training [12], with some multimodal programs including dietetic and psychological counselling [8].

A challenge for improving multimodal prehabilitation and postoperative outcomes in all colorectal surgery patients is that, since little is known regarding patients’ perceptions or experiences with presurgical care, there is little direction for developers of effective prehabilitation to follow. Possible barriers to prehabilitation could be the high patient burden of engaging separately with all the multi-disciplinary team providers [13].

The missing element of published research regarding multimodal prehabilitation in abdominal surgery is that patients are not heavily engaged in the program’s design. A few qualitative studies in Holland and in Eastern Canada report patient experiences of preoperative care [12, 14–16], but there is still little understanding of the gap between current prehabilitation programs and the health priorities of colorectal surgery patients (with and without cancer), and whether their concerns are addressed from a person-centred care approach. This omission affects successful uptake of interventions into clinical practice which ideally involves patient-partners in co-creating intervention-specific knowledge and co-planning program development [17].

This paper aims to describe experiences and explore preferences for multimodal prehabilitation (e.g. exercise, diet and psychosocial stress) among patients waiting for a national referral centre for complex colorectal surgery in Western Canada. This research will fill knowledge gaps through insights from patients on their experiences and preferences regarding surgical prehabilitation to advise a patient-centred multimodal prehabilitation program.

## Methods

### Research design

We designed this qualitative, descriptive study using three focus groups (FG) since FG methodology allows researchers (1) to explore the specific topic of multimodal prehabilitation (e.g. improving diet, exercise, limiting alcohol and smoking, supporting psychological factors) in abdominal surgery, and (2) to understand preoperative experiences of supportive care among colorectal surgery patients.

### Participants

We purposively sampled patients who received abdominal surgery for colorectal conditions at St. Paul's Hospital Colorectal Clinic (April 2017-April 2018). This 1-year period was selected to include patients who were actively waiting for surgery so as to learn about current experiences of, and preferences, for preparing for colorectal surgery. Consecutive patients in this period were eligible for this study to obtain a sample of 8 to 10 patients.[18] Exclusion criteria were: under 18 years, limited proficiency in English and, for ethics reasons, living close to the location (≤ 50 km). Eligible patients were recruited by health professionals working in St. Paul's Hospital Colorectal Clinic – that includes a surgeon who is a national expert and hospital lead in Enhanced Recovery After Surgery (peri-operative and post-operative support) – through a letter of invitation. Following consent to contact, a research assistant phoned the patient to provide further information and project goals. Interested patients received comprehensive study information in writing. Twelve recruited patients were diagnosed with cancer (4 had colon and 8 had rectal-related cancer) and seven with non-cancer conditions (ulcerative colitis, Crohn’s disease, serrated adenomatous polyposis syndrome, etc.). All patients came from white-European backgrounds, except one who was Asian; thereby excluding patient experiences from individuals who identify as Indigenous, Black (Africans, African-Americans, etc.), Hispanic, Southeast Asian and South Asian who are also patients in this provincial referral centre.

### Recruitment

The study team selected patients according to age, sex, and indications for surgery to attend one of three FG meetings [18], and selected. Our purposive sample had patients who had non-cancer abdominal conditions (*n* = 6 out of 75 invited) for one focus group and people who had colon and/or rectal cancer (*n* = 5 out of 150 invited) for another. Our third focus group included both patients with cancer (*n* = 6) and other abdominal conditions (*n* = 2), so as to provide an opportunity to verify our preliminary findings from the first two groups.

### Data collection

The FGs took place at a meeting room at St. Paul's Hospital, and lasted between 90 to 120 min. A skilled moderator (CY) and assistant (LM), who had limited knowledge of this specific topic, guided the FGs using open-ended and probe questions (supplementary Table S1). Two general questions were used to capture a range of experiences (‘what were your challenges while you were waiting for surgery?) and views of preoperative support for exercise, diet or emotional stress/anxiety (what are your preferences for better preparing for surgery?). In order to not prejudice the participants, we chose to use words like “time leading up to your surgery” or “as you were waiting for your surgery” rather than using the specific term of “prehab”.

### Data analysis

Tape-recorded FG discussions were transcribed verbatim and transcripts underwent multiple iterations of data review and analysis. Each transcript was read and coded (CY, LM, RW) line-by-line for descriptive experiences. Initial data analysis (CY) enabled member-checking of early emergent parent codes and themes and also further exploration and analytic questions of patients’ experiences and views of preoperative supportive care in the third FG. Data analysis explored the experiences of preparing for colorectal surgery and preferences for a program to better support patients while they are waiting. The analysis team (RW, CY, LM, AC) inductively developed a shared codebook for selective coding using 19 (sub)parent codes developed after initial open-coding. Codes were organised into categories (RW) and discussed (RW, AC) in order to show patterns in semantic content, theorise their significance and broader meanings and implications [19]. Emergent themes were iteratively developed from the exploratory categories until thematic saturation was achieved after careful review of relevant constructs from patient-centred care and chronic disease management literature. We used the pile-and-sort methodology to group the list of parent codes into related themes and to contextualise relationships between the codes and the concepts they might represent [20], and constructed latent labels to reflect underlying concepts and existing literature [21] (supplementary Table S2). Each stage of analysis was reviewed by the study lead (AC) who was not involved in data collection (CY, LM, SL). RW re-read the transcripts to selectively search for data illustrative of identified themes. Data analysis was done using NVivo 11.4.3 software.

## Findings

**Fig. 1:**
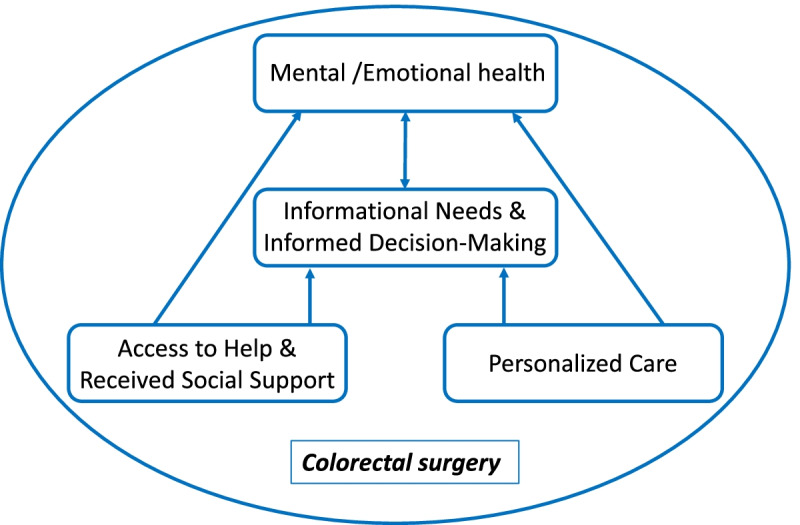
Conceptual model of identified themes, showing how mental and emotional health (theme 4) and informed decision-making, including informational needs (theme 2) were prominent concepts reinforced and determined by both access to and received social support (theme 1), and personalized care (theme 3); with mental health and informed decision-making mutually reinforcing one another in the setting of colorectal surgery

Table 1 shows some key characteristics of our study participants in terms of demographics, socioeconomic status, and access to healthcare. Our results revealed that most patients had been given a short one-page surgical preparation booklet, but did not receive structured care prior to surgery and especially for the non-cancer patients waiting for colorectal surgery. Four main themes about preoperative experiences and programming preferences, common to both cancer and non-cancer abdominal patient, were generated from the data analysis: 1) access to help and received social support, 2) informational needs and informed decision-making, 3) personalized care, and 4) mental and emotional health. Our thematic map shows how mental and emotional health (theme 5) and informed decision-making, including informational needs (theme 2) were prominent concepts reinforced and determined by both access to and received social support (theme 1), and personalized care (theme 3); with mental health and informed decision-making mutually reinforcing one another in the setting of colorectal surgery (Fig. 1).

**Table 1:** Patient characteristics

Characteristic	Cancer patients (*n* = 11)	Non-cancer patients (*n* = 8)	Overall (*n* = 19)
Female, n (%)	6 (55%)	6 (75%)	12 (63%)
Age (years), median and range	63 years (33–72)	55 years (31–72)	58 years (31–72)
Married, n (%)	7 (64%)	6 (75%)	13 (68%)
Bachelor education or above, n (%)	4 (36%)	5 (63%)	9 (47%)
High income level (> $100,000), n (%)	6 (54%)	5 (63%)	11 (58%)
Full-time employed, n (%)	3 (27%)	5 (63%)	8 (42%)
Non-smoker	7 (64%)	8 (100%)	15 (79%)
Received pre-surgery booklet^a^, n (%)	10 (91%)	6 (75%)	16 (84%)
Obtained pre-operative support from a health professional, n (%)	5 (45%)	1 (13%)	6 (32%)

^a^ The pre-surgery booklet is a printed copy of the clinic’s patient information and education about colorectal surgery (10–12 pages), with one page of information on smoking, alcohol diet, and exercise

### Theme 1: access to help and received social support

Having access to help and receiving positive support from one’s social ties, both informational and emotional support, before surgery were important (Table 2). Peers who had gone through similar surgeries were always seen as a valuable source of positive support in terms of both practical information and mental/emotional wellbeing. However, experiences of support from family, friends, and healthcare providers were mixed. The support provided by peers included not only information and tips about the surgical process but also emotional support, particularly pertaining to the unique challenges related to bowel surgery that may be difficult to share with close family and friends.

**Table 2:** Illustrative quotes for Theme 1 on patients’ experiences and perspectives on preoperative care in colorectal cancer and abdominal surgery

Theme	Examples of Quotes
Access to Help & Received Social Support	“I can’t talk about the fact that I need to go and buy adult diapers to my best friend, like I just don’t want to do that. To have a person who can hear all the nitty gritty and it doesn’t shock them, and they can empathize. That was huge” (FG1, P5) “Somebody that’s gone through it rather than an expert (FG3, P3) “I don’t know what I would have experienced if I hadn’t had the support from my doctors and my surgeons. They were all great” (FG1, P2)
	“[M]y doctor told me when I was going in that I was going to the bathroom 10–15 times a day and bleeding like crazy, he just tells me I just got diarrhea, don’t worry about it. Two weeks later I was still doing it. So, the doctors should be taking things a little more serious to what is going on” (FG1, P3)
	“[J]ust the sheer number of appointments like holy cow it’s a job to have…having cancer is a job.” (FG2, P2) “[I]t’s not them calling us, it’s when we get the urge, we have somebody to call because often times they are calling and it’s not convenient or we are forgotten what it is that we wanted to talk about” (FG2, P4) “[T]he accessibility. It also has to work around the time you have to get away from work if you are working. Do you have to come downtown to do it or can I go to a clinic close by? The more accessible it is the more likely you are to go.” (FG3, P3) “Once you know what you have to deal with you just deal with it. But it’s the unknown, once you know what you got to deal with, the outcomes and stuff like that. But you might have questions that come up, and if you have a phone number that you can phone and just answer your questions, that would be good, and move on.” (FG2, P4) “[R]eally you need somebody there to carry the mail for you” (FG2, P3)

Multiple suggestions were made regarding how to integrate peer support into care, including the promotion of support groups, patient networks to connect preoperative patients with past surgical patients, and group seminar sessions with past patients to help reduce their sense of isolation. Patients preferred to receive structured support at the time of diagnosis as this was seen to positively support mental health by alleviating some uncertainties.

In terms of family and friends, patients had divided opinions on their helpfulness prior to surgery. Opinions ranged from liking the concern and care people gave to finding it a burden to keep friends calm once they found out about the diagnosis. Similarly, healthcare providers were either a source of support or frustration based on their attitudes. Patients found it helpful when healthcare providers addressed their questions and gave them explanations, but had more confusion and uncertainty when information conflicted between providers or was full of medical jargon.

Patients also felt healthcare services consumed a lot of time, similar to full employment, and would benefit from being accessible on an ongoing basis or when needed. Patients were particularly interested in being connected with a person when they needed support and information such as for nutrition and mental health.

Patient experiences highlighted potential timing problems that could affect prehabilitation programming. These included: work-schedules conflicting with in-person appointments, the urgency of patient medical conditions limiting lifestyle changes, and the need to understand post-surgical nutrition needs earlier in the process. While many patients emphasized the importance of being able to call for support rather than being phoned at an inconvenient time, one patient preferred being contacted since it triggered more pro-action (and helped avoid denial) about their health.

### Theme 2: informational needs and informed decision-making

Patients wanted at least a baseline amount of information about preparing for colorectal surgery, including information on different risk factors to make their own decisions about how they might engage with a multimodal prehabilitation program (Table 3). However, individuals varied widely on their informational needs for achieving informed decision-making. A number of patients felt a responsibility to take control of their own health and wanted to be independent, but several others preferred to be given professional guidance, particularly supervised exercised. Overall, patients generally felt that there was a lack of information given to them, but a couple patients preferred to have less information given to them. One suggestion to accommodate both parties was to offer the information about preoperative self-management so that it was available for patients who wanted to look at it.

**Table 3:** Illustrative quotes for Theme 2 on patients’ experiences and perspectives on preoperative care in colorectal cancer and abdominal surgery

Theme	Examples of Quotes
**Informational Needs and Informed Decision-making**	“I’d do it myself, but I’d like somebody to say that if I did my exercises and stuff I would be much better physically prepared for the operation than if I just laid around and smoked all the time” (FG1, P4) “I’m the kind of guy that likes information and I didn’t get sufficient information…I could have benefitted by a whole lot more of information myself. For other people, too much information can make you even more stressful but I didn’t have sufficient information” (FG3, P7)
	“So I had hip replacement surgery and before I had it, they said ‘you should lose 40 pounds and you should do these exercises.’ Well I did it 100%, I lost the 40 pounds, I did the exercises because it was specific and I could understand the benefit. But with this colon cancer, I just don’t see any…you have to tell me something more” (FG3, P8) “I don’t think it’s a doctor-hospital, not our province’s medical responsibility to hold our hands to exercise” (FG1, P6) “I think to have a set program that could be delivered at a location by a physical trainer… would be perfect. Getting the information… I wouldn’t be inclined to do it myself. I would be more likely to just carry on with my own routine that I use. So, having to go in, having to fulfill a 4-week or 6-week program 2 to 3 times a week would be more advantageous I think” (FG2, P3)
	“I said, “I’m not trying to sound like an idiot here guys, but can someone tell me what the heck you are talking about”?” (FG2, P2) “Not just a little pamphlet here’s a pamphlet, like I said before it doesn’t tell me anything. It’s the generic thing they tell everybody, half the stuff that was in the pamphlet wasn’t happening to me. Again, somebody to say, “here’s the things that are going to happen and this is what’s going to be after”” (FG1, P4)
	“[I]t’s great to actually see someone do something and say, “okay I know I am doing it right” and not for example increasing my risk of getting a hernia” (FG2, P2) “I would put on more [materials for support] except the nutrition or support, exercise, I would say well-being and helping. Spiritual well-being and helping is more important” (FG2, P5) “a resource, somebody you could pick up the phone and call and ask some of these questions, to put your mind kind of at ease” (FG2, P4)

In terms of preferences for structured preoperative support, patients wanted both written information and support from a person who is trained or has lived experience. The majority of people felt the surgical booklet provided was insufficient and raised more questions, but some had found it useful. Patients felt in-person delivery was particularly helpful for emotional support, personalising care, and ensuring safety. And, although the Internet could be a useful source of information, patients wanted professional help in differentiating good and bad information they found online, or be given a list of trustworthy information.

### Theme 3: personalized care

Patients often desired tailored support and information to help prepare for colorectal surgery, particularly in terms of prehabilitation exercise, and many expressed the need for clear explanations on how proposed prehabilitation interventions for exercise and nutritional support would specifically benefit surgical outcomes and recovery (Table 4). One patient suggested being presented with the different options for psychosocial supports available, such as counselling or peer support, so presurgical patients could choose the type of support desired based on their individual struggles. Although personalization of care was clearly important for patients, they also agreed there were aspects of prehabilitation that were generally helpful to everyone.

**Table 4:** Illustrative quotes for Theme 3 on patients’ experiences and perspectives on preoperative care in colorectal cancer and abdominal surgery

Theme	Examples of Quotes
**Personalised Care**	“So I had hip replacement surgery and before I had it, they said ‘you should lose 40 pounds and you should do these exercises.’ Well I did it 100%, I lost the 40 pounds, I did the exercises because it was specific and I could understand the benefit. But with this colon cancer, I just don’t see any…you have to tell me something more” (FG3, P8)
	“I would say not a need for any. I have managed through diet and exercise for more than two decades. So just knowing my own body.” (FG1, P6)
	“I don’t feel like you get specific nutritional care from a hospital nutritionist (P1: Or the food). Yeah, but even aside from that there are bowel disease and these things that we’re dealing with, there’s a real holistic component to them and there is a real nutritional component to them and its super specific. Until that’s sort of learning process seems to change a little bit, I don’t know how they actually offer a service that has value to us individually” (FG1, P3) “Not just a little pamphlet here’s a pamphlet, like I said before it doesn’t tell me anything. It’s the generic thing they tell everybody, half the stuff that was in the pamphlet wasn’t happening to me. Again, somebody to say, “here’s the things that are going to happen and this is what’s going to be after”” (FG1, P4) “you might have questions that come up, and if you have a phone number that you can phone and just answer your questions, that would be good, and move on.” (FG2, P4) “Because mine was a non-cancer and it was like a prophylactic scenario I knew exactly what the surgery was going to be, all the steps in the surgery. Everything about it. But when I went to the pre-admission clinic I got on some sort of treadmill where they were talking to you in a rope sort of way because I’m sure they have to tick boxes off to make sure all the general information was given out the same way but it didn’t feel like it was customized to me.” (FG3, P4) “there’s a bit of a “one size fits all” thing. We can talk about this later where you know on the post-op I felt that this is written for an “overweight smoker”. Do I really have to wait 6 weeks before I pick up the cat? It just didn’t make sense to me.” (FG3, P1) “It would be nice to be able to pick up the phone if you have a question. But everyone is so different for everyone else so one size can’t really fit all in this situation.” (FG3, P3)

Personalized care was also shown by the widely varying preferences for the amounts and delivery of prehabilitation. There were strong differences regarding dietary support based on the type of bowel disease. Most patients with colorectal cancer expressed no nutritional difficulties and thought general nutritional support could be helpful. By contrast, patients with more chronic bowel conditions (e.g. inflammatory bowel disease) had more difficulties and felt the available dietetic counselling was too generic to be helpful and that they knew their own bodies well enough to self-manage. Nonetheless, this latter group of patients expressed interest in dietary prehabilitation if the educational support could be more specific and personalised as well as include dietary recommendations for healthy alternatives.

### Theme 4: mental and emotional health

Patients experienced many negative emotions during the preoperative period such as denial, confusion, stress, isolation, and anxiety (Table 5). In addition to challenges associated with their medical conditions and upcoming surgeries, factors contributing to decreased mental and emotional health included the uncertainty of surgery schedules, the possibility of last-minute surgery cancellations, and the struggle with planning and moving forward with life when surgical outcomes were still unknown. Thus, all patients wanted more support with mental and emotional health, which they felt was not adequately addressed. For many, having support for mental and emotional health was closely tied to their physical well-being, particularly through nutrition.

**Table 5:** Illustrative quotes for Theme 4 on patients’ experiences and perspectives on preoperative care in colorectal cancer and abdominal surgery

Theme	Examples of Quotes
**4. Mental and emotional health**	“[S]omeone telling us now you are at this point, what’s next, what’s next, what our system is. What situation you’ll be treating, how good is your surgeon, how many people will be recovered from this. It gives you confidence. You feel much better. So, I hope all this is done at the very beginning.” (FG2, P5) “Yeah, its stress…. apprehension is the wrong word. It’s just a disappointment like you feel like you’re just sitting there, and you can’t go forward because you don’t know what forward is yet.” (FG2, P1)
	“[W]ith the stress that you’re under, you’re not thinking properly, you’re not eating properly” (FG3, P6)
	“[I]t would have helped to release the stress so that you’re busy, focused on something else, so that you can control versus something that you couldn’t control” (FG3, P6) “[Mental health is] not addressed here at all and you’re really left floundering” (FG1, P1) “She spent a lot of time on mental health with me too, really talking a lot about that, which was really important” (FG1, P4) “have that person’s contact information so you could potentially call them if you’re in a crisis or if you need to talk to someone” (FG1, P3) “Fear.” (FG2, P1) “and then the anger thing, right? You sort of do the balance of trying to continue working and there’s a whole lot to take and also the fear of the unknown” (FG2, P2) “Dire messages from my doctor and surgeon, so I kind of had that in the back of my head.” (FG3, P1) “it was the bog-standardness of my experience in the pre-admission clinic which created a huge emotional burden (FG3, P4) “a resource, somebody you could pick up the phone and call and ask some of these questions, to put your mind kind of at ease” (FG2, P4) “I would put on more [materials for support] except the nutrition or support, exercise, I would say well-being and helping. Spiritual well-being and helping is more important [….] if the mind calms the bad things this is really helpful” (FG2, P5)

Patients found that keeping a positive mindset, peer-support groups, and meditating helped them cope with their negative emotions. Exercise also boosted confidence, increased morale, and helped patients focus on controllable aspects of their lives.

Patients gave multiple suggestions for how psychosocial support for mental and emotional health could be implemented in the preoperative phase. These included facilitating new and past patients with similar surgeries to connect and share feelings and useful tips, offering trained counsellors for dealing with the specific challenges of bowel surgery like wearing an ostomy bag, or hosting a seminar with both healthcare providers and past patients to gain more information and help alleviate apprehension.

In summary, our data revealed four themes related to preoperative care experiences and preferences in colorectal surgery. Mental health emerged as a particularly strong and pervasive theme, with aspects of all three other themes linking to it.

## Discussion

Aligning healthcare delivery to improve patient health status is an important objective of surgeons, healthcare administrators and funders. Strengthening evidence supports prehabilitation as a valuable tool for achieving these objectives. However, research on prehabilitation interventions is needed in order to strengthen the interventions’ individual or combined components in order to achieve patients’ objectives for their surgical episode and postoperative health. This qualitative study contributes to the literature by finding that there is significant room to improve multimodal prehabilitation for colorectal surgery patients, including convenience, expert-vetted information, and personalization.

Overall, patients experienced inadequate preoperative support and felt mental health was especially important but rarely addressed. This unmet need and preoperative care gap is corroborated by other studies finding that fear and uncertainty are prominent negative emotions experienced by other preoperative patients [22], further emphasizing the need to incorporate mental health as a focused aspect of multimodal prehabilitation.

Perceived deficiencies in the healthcare system can also enhance anxiousness and frustration [23], highlighting links to our study’s Theme 1 of access to and receipt of support, particularly conflicting information from healthcare providers. For example, delayed access to surgery can lead to a feeling of lack of control and psychological stress [23]. Poor mental health in abdominal surgery patients has numerous ramifications, including maladaptive coping strategies of self-control, self-blame, and escape that are linked to decreased quality of life [24]. Recent research shows that preoperative depression is linked to longer lengths of stays among colorectal surgery patients in British Columbia [25].

Integrating peer support into prehabilitation programming could improve both patient mental health and postsurgical outcomes. Patients highlighted how peer support helped alleviate feelings of isolation, which is concordant with previous research on preoperative rectal cancer patients [22]. Greater social support has also been linked to better survival, remission and quality of life among patients with colorectal cancer and non-cancer [10, 22, 24]. One mechanism linking peer support to better patient outcomes is a sense of togetherness and accountability, and motivation for lifestyle changes [10]. By contrast, patients had mixed experiences of non-peer support, with negative effects echoing other studies that found patients either worry about their condition affecting family/friends or worry about their own poor outcomes when a family/friend had a similar condition [9, 26].

Although healthcare providers often provided positive support, patients experienced frustration when providers did not adequately listen to patient concerns, used a lot of medical jargon and/or contradicted one another. This is consistent with earlier studies [10, 23, 27], reporting that colorectal cancer survivors received conflicting information about lifestyle advice, and that limited explanation or scientific information can cause confusion and lead to feelings of vulnerability. Clear and consistent information from healthcare providers is therefore essential given their influence on patients’ behaviour changes [12]. Particularly important to patients is an explanation of the link between preoperative lifestyle changes (each prehabilitation component) and surgical outcomes so they could make informed decisions about accessing preoperative care, which others also report [9, 12].

Importantly, patients in this study varied widely in the amount, type, source, frequency and format of information on lifestyle advice they wanted. More information was seen to decrease fear and improve confidence for some patients while less information might decrease fear for others, as reported elsewhere [9]. Non-cancer abdominal patients strongly preferred personalized dietary information and expressed frustration and disappointment with the usual care of generic dietary advice based on Canada’s Food Guide. Limited research on rectal cancer patients supports the patient-reported benefits of tailored information, including better shared decision-making and reduced postoperative anxiety [28]. Thus, any future preoperative care information needs to better engage patients and include different support options to account for the range of patient needs.

Preference differences for accessing preoperative supportive care further underscored the need for personalising multimodal prehabilitation. Many respondents wanted to access self-management support at any time which could allow patients more time to process health information and receive help whenever they needed it [29]. Nevertheless, some preferred more active support, as receiving regular calls for self-management support could contribute to patients’ sense of being emotionally supported and provide a structured opportunity for more information [12]. Having flexible access would also help address known barriers to participation in disease management programs such as transportation and time constraints [12]. Personalizing preoperative care is important because it is patient-centred and ensures more consistent alignment with diverse patients’ preferences and/or their physical capabilities, thereby enhancing adherence [30].

### Methodological considerations

Our interpretations are drawn from three focus groups of nineteen patients who received colorectal surgery from a single hospital in Vancouver, Canada, and were mostly one ethnicity of relatively high socioeconomic status. This may limit the study from capturing the full breadth of patient experiences of preoperative care and preferences for multimodal prehabilitation, which future studies should consider in their design. This study is inherently limited by the FG methodology that can increase the risk of social conformity. However, our use of multiple FGs, using member-checking with the third which had more mixed patients, may have helped reduce the impact of this known psychosocial process.

A strength of this qualitative study using the focus group methodology is that we gained a broad range of views and collective meaning on the specific burgeoning topic of surgical prehabilitation in a short period of data collection. In exploring this new topic, our method allowed us to reveal an unmet need for preoperative support that addresses multiple health domains of colorectal surgery patients, especially emotional/mental health. There was group consensus on patients waiting for colorectal surgery to have a single source of professionally-vetted information on lifestyle advice and many also needing a structured approach to help them with simple strategies to stay healthy regarding exercise, diet and stress/anxiety.

### Practice implications

The evidence for effective multidisciplinary prehabilitation interventions for colorectal surgery is strengthening [5]. A primary implication of this study is that patients (and their caregivers) have a role as partners in the development of future multimodal prehabilitation, which should be made as flexible as possible to account for the range of needs. These results have informed our current patient-oriented research project to test the feasibility of a new web-based multimodal prehabilitation program. This involved the co-creation of a patient-oriented website comprising evidence-based professionally-vetted information and patient self-management materials to better support patients in preparing for colorectal surgery (prehab.cheos.ubc.ca), and included: information on the benefits of prehabilitation, the evidence for surgical outcomes improving each lifestyle factor (e.g. smoking, alcohol, diet, exercise, emotional stress), practical recommendations and available resources and links for further self-management support. The second implication for practice is that surgeons have a critical role to provide clear and consistent information about the benefits of multiple lifestyle changes that help patients achieve better outcomes. Doing so appears essential for better patient mental health and shared decision-making, and may also improve prehabilitation effectiveness. Finally, the third implication for practice and policy (including funding) is the provision of psychosocial support in colorectal surgery.

## Conclusion

We found an unmet need for preoperative support for patients waiting for colorectal surgery. There was a strong patient preference for improving mental/emotional health and wellbeing and need for personalised and reliable information that clarified the role of lifestyle modification as preoperative care could improve for surgical outcomes. Since patients’ concerns and needs varied considerably, some personalization and flexibility are needed in future interventions so that patients could choose the amount and type of preoperative support they received. This study can help inform future research, policy and delivery of patient-centred multimodal prehabilitation for colorectal surgery, and highlights that multimodal prehabilitation should incorporate patient preferences.

## Supplementary Information

**Additional file 1:** **Supplementary Table S1.** List of common guiding questions used in the focus groups to explore patient experiences and preferences for prehabilitation in colorectal surgery. **Supplementary Table S2.** Final codebook developed during data analysis. 

## Data Availability

All relevant data are provided in the manuscript and appendices. Original transcripts could not be deposited in the public domain due to confidentiality of personal information.
