# Supplementary material for: Preparing for colorectal surgery: a qualitative study of experiences and preferences of patients in Western Canada
Source: BMC Health Serv Res. 2022 Jun 1;22:730. doi: 10.1186/s12913-022-08130-y (PMC9161453; doi:10.1186/s12913-022-08130-y)
Supplement: Supplementary file 1 — Additional file 1: Supplementary Table S1. List of common guiding questions used in the focus groups to explore patient experiences and preferences for prehabilitation in colorectal surgery. Supplementary Table S2. Final codebook developed during data analysis. [file 12913_2022_8130_MOESM1_ESM.docx]

**Supplementary Table S1. List of common guiding questions used in the focus groups to explore patient experiences and preferences for prehabilitation in colorectal surgery**

| Challenges during surgical wait-time | First off, I’d like to ask you about the challenges you may have encountered or experienced while you were waiting for your surgery. Specifically, we would like to ask you about challenges that were related to physical activity or exercise, diet and eating habits, and emotional health. If you have any other challenges to share with us, we can talk about those as well.   - 1. Let’s start by talking about physical activity and exercise.   2. What about diet and eating habits?   3. What about emotional and mental health?   4. Were there any other challenges you encountered during this time? |
| --- | --- |
| Feelings, perceptions, and preferences for prehabilitation programs | Next, we’ll be asking you about if you would have liked to receive a program offered by the hospital to help you solve or address these challenges. By program, we mean a structured set of information, services, strategies, or materials. The main purpose of this program would be to help you better prepare for your surgery.  Let’s start by talking about exercise or physical activity.   - - 1. In a scale between 1 – 10, how likely were you to participate in exercise and physical activity offered to you during your surgical wait time.     2. How would you feel about receiving help with being more physically active or to do more exercise during your surgical wait time?     3. What kind of services, supports, or information would you have liked to receive about physical activity or exercise?     4. Would you prefer to come to the hospital for exercise, or exercise at home?     5. Would you like someone who you can contact for advice and information about exercise during your surgical wait time?     6. Would you like endurance exercise, strengthening exercise, or both?     7. How often would you exercise during this time? |
|  | Next, let’s talk about nutrition and eating habits.   1. In a scale between 1 – 10, how interested would you have been in receiving help for your nutrition and healthy eating during your surgical wait time? 2. How would you feel about receiving help with your nutrition and eating healthier? 3. What kind of services, materials or supports would you have liked to receive to help you with this? 4. Would you like to come back to the hospital for nutrition counselling? |
|  | Now, let’s talk about emotional and mental health.   1. In a scale between 1 – 10, how interested would you have been in receiving help with your emotional and mental health? 2. If you were feeling anxious or depressed during your surgical wait time, how would you feel receiving help with your emotional and mental health during your surgical wait time? 3. What kind of services, support, or materials would you have liked to see to help with your emotional and mental health during your surgical wait time? |
|  | What other elements of services, support, or materials would you like to see provided by the hospital to help you prepare for your surgery? |
| Perspectives on the health coach model for prehabilitation | Finally, how do you feel about receiving help in the form of an education session by someone (“Pre-Surgical Health Coach”) after you have decided to have surgery at St. Paul’s Hospital? This education session might include information and guidance on how to stay active, eat healthier, and to help with your emotional and mental health during your surgical wait time.  What specific components would you like to see included in such education sessions by a Pre-surgical Health Coach?   - - 1. What do you think about weekly telephone calls offered by the life coach?     2. What do you think about a diary to log your physical activity and diet?     3. What do you think about relaxation music to help with your emotional and mental health? |
| Closing | To sum up, can you please tell me about anything else that you can think of that would help you better prepare for your surgery? |
|  | Is there anything else that you would like to add that wasn’t covered today? |

**Supplementary Table S2. Final codebook developed during data analysis**

| **Themes** | **Parent Code** | **Description** |
| --- | --- | --- |
| 1. Access to Help and Received Social Support | 1. Peer Support Systems (n=33) | Patient views on necessity/importance of help from other current/past surgical patients as well as willingness to help other pre-surgical patients |
|  | 2. Health Care Provider Support (n=121) | Perceptions of care and support from healthcare providers (e.g., doctors, nurses, nutritionists, surgeons, etc.), including views of care from an interprofessional team |
|  | 3. Family Relationships (n=22) | Impact of patient disease/surgery on family members and the impact of family members on pre-surgical patients |
|  | 4. Social Contact (n=22) | Impact of patient medical condition/surgery on others/relationships with others (e.g., friends, coworkers, bosses, etc.) and impact of others on patients |
|  | 5. Time Burden (n=15) | Patient views on the amount of time involved with receiving healthcare services or surgery preparation |
|  | 6. Timing of the Healthcare System (n=53) | Patient perceptions on the timing of the healthcare system (e.g., surgery wait times, notifications of getting surgeries cancelled when other patients are bumped, etc.) |
|  | 7. Accessibility of Ongoing Support (n=26) | Refers to pre-surgical patient perceptions of the ease in which they can reach out for support throughout the entire surgical process (including mental, emotional, or physical help in between appointments with HCPs) |
| 2. Informational Needs and Informed Decision-making | 8. Taking Responsibility for Own Health (n=40) | Patient perspectives on or measures taken for improving own health and views on the necessity of outside guidance/help for health-related measures prior to surgery |
|  | 9. Information Resources (n=42) | Views of non-human resources such as pamphlets, apps, online articles, and videos patients can use for informational purposes |
|  | 10. Uncertainty (n=32) | The uncertainty and unknown surrounding aspects of patients’ health and healthcare (e.g., not knowing about the after-results of surgery until it is completed, conflicting information, etc.) and its impact on patients |
|  | 11. Lifestyle Adjustments (n=38) | Patient views on their medical conditions/surgery and associated challenges with respect to lifestyle and day-to-day life as well as resulting measures taken due to them |
|  | 12. Quantity of Information (n=142) | Refers to the amount of information patients know. Also refers to patient views on the quantity of knowledge they have and gaining more knowledge (e.g., ease, necessity, etc.) |
|  | 13. Monetary Cost (n=13) | Refers to patient perspectives on the financial cost associated with their medical condition/surgery (including pre-op and post-op costs) |
| 3. Personalized Care | 14. Personalized Care (n=47) | Patient views regarding current presence (or lack) of personalized care as well as having care more catered to their individual needs |
| 4. Mental/  Emotional Health | 15. Mental/  Emotional Health (n=174) | Emotions and mental states (e.g., depression, anxiety, etc.) that patients experience before or immediately after their surgeries and their views on receiving help for this aspect |
|  | 16. Relaxing the Mind (n=21) | Patient views on the importance of mindset and how to achieve a more relaxed mindset (independent of healthcare provider assistance) |
| All (1-4) | 17. Physical Activity (n=126) | Pre-surgical patient views on exercising and exercise habits. Also refers to patient perspectives on guidance/support for physical activity |
|  | 18. Nutrition (n=98) | Refers to patient changes (or lack thereof) in diet or nutrition associated with the surgical process/medical condition, nutritional support from HCPs, and other nutrition-related resources, as well as patient perspectives on all of these aspects |
